# A flexible five‐channel shielded‐coaxial‐cable (SCC) transceive neck coil for high‐resolution carotid imaging at 7T

**DOI:** 10.1002/mrm.28215

**Published:** 2020-02-12

**Authors:** Thomas Ruytenberg, Andrew Webb, Irena Zivkovic

**Affiliations:** ^1^ C.J. Gorter Center for High Field MRI Department of Radiology Leiden University Medical Center Leiden the Netherlands; ^2^ Carle Foundation Hospital Urbana IL USA

**Keywords:** 7T MRI, carotid imaging, neck coil, ultra‐high field

## Abstract

**Purpose:**

Imaging the carotid arteries at 7T ideally requires a flexible multichannel array that allows B1‐shimming and conforms to different neck sizes. The major challenge is to minimize coupling between closely spaced coils and to make the coupling relatively insensitive to loading conditions.

**Methods:**

We have designed a five‐channel flexible transceive array composed of shielded‐coaxial‐cable coils placed on the anterior part of the neck and conforming to the anatomy. In vivo imaging of the carotid arteries in three subjects has been performed.

**Results:**

The measured noise correlation matrices show the decoupling level between the individual elements to be −12.5 dB and better. Anatomical localizer imaging of the carotids shows both carotids in every subject well visualized after B1‐shimming. In vivo black‐blood, carotid images were acquired with very high in‐plane spatial resolution (0.25 × 0.25 mm^2^) with clear depiction of the vessel walls.

**Conclusions:**

The flexibility of the proposed coil has been demonstrated by imaging subjects with different neck circumferences. To the best of our knowledge, the in‐plane resolution of 0.25 × 0.25 mm^2^ is the highest reported at 7T.

## 1 INTRODUCTION

There is a high level of interest in using noninvasive MRI for vascular imaging in atherosclerosis, which is a systemic disease affecting the intermediate‐ and large‐sized arteries.^1^ In addition to chronic problems associated with the disease, the presence of so‐called “vulnerable plaques” presents an acute condition that may lead to myocardial infarction or stroke.^2^ High‐resolution imaging of the carotid arteries can identify regions of stenosis and abnormal arterial blood flow, and information on the components of a particular plaque, such as lipids within the core, the nature of the fibrous cap, and any intraplaque hemorrhage can be obtained if the spatial resolution, signal‐to‐noise (SNR), and contrast‐to‐noise (CNR) are sufficiently high.

Given the latter requirements, the use of very high field (7 T and above) MRI scanners would appear to be promising for carotid artery vessel wall imaging.^3^ However, there have been relatively few very high field studies of carotid artery imaging since specialized multielement transmit/receiver or transceive hardware needs to be developed. The first demonstration at 7T was a rigid 2 × 4 loop transceive array published by Kraff et al,^4^ which covered both arteries and utilized a commercial 8‐channel multitransmit architecture from Siemens. Images were acquired from a healthy volunteer and also a patient with extensive stenosis of an ulcerating plaque. The first comparison of image resolution and contrast at 7 T vs. 3 T was presented by Kroener et al,^5^ who used two slightly flexible loops, one for each carotid artery, an architecture that was limited at that time by having only two available transmit channels on the Philips platform. Images acquired from healthy volunteers included T1‐ and T2‐weighted anatomical scans, as well as bright‐blood methods, and showed statistically significant improvements in vessel wall SNR and lumen wall CNR at 7 T. Measurements of the luminal wall and vessel wall cross‐sectional areas were the same at the two field strengths. A significantly different type of approach was used by Koning et al^6^ who used two radiative antennas as transmit elements, and two receive arrays (one for each carotid) each consisting of 15 small (~2 cm diameter) loops. The authors reported an increase in SNR of a factor‐of‐two compared to scans acquired on a 3 T scanner. In order to improve the efficiency of suppressing signal from inflowing blood, Papoutsis et al^7^ introduced a coil architecture consisting of a 2 × 2 transmit array (2 placed at the level of the carotid bifurcation and the other at the lower neck) and a 2 × 2 receive array placed at the level of the carotid bifurcation. This geometry enabled black‐blood imaging to be performed using the delay alternating with nutation for the tailored excitation (DANTE) method for in‐flow suppression.

In terms of coil design for carotid vessel wall imaging, there are many improvements that need to be made for practical patient use. The main one is that the coils need to be adaptable to a large number of different neck sizes, particularly when considering patients with a high body mass index. This means that the coil arrays must be very flexible, and the interelement decoupling must be relatively insensitive to changes in their geometry (i.e., bending and flexing) as well as to the degree of loading in terms of being close to a smaller or larger degree of lipid or muscle. To address these issues, in this work we have designed a flexible five‐element neck array with each element consisting of a shielded‐coaxial‐cable (SCC) coil.^8^ The SCC coil is an element that can be constructed from very flexible thin coaxial‐cable, does not require distributed lumped element capacitance within the structure, and has been shown to have a high degree of intrinsic decoupling between elements as well as a lower dependence of tuning and decoupling with respect to geometric deformation than for conventional surface coils.^8^ Here, we assess the use of such an array in obtaining very high spatial resolution (0.25 × 0.25 mm^2^ in‐plane) black‐blood images of the carotid arteries in healthy volunteers with different neck sizes.

## 2 METHODS

### 2.1 Coil design and fabrication

**Figure 1:**
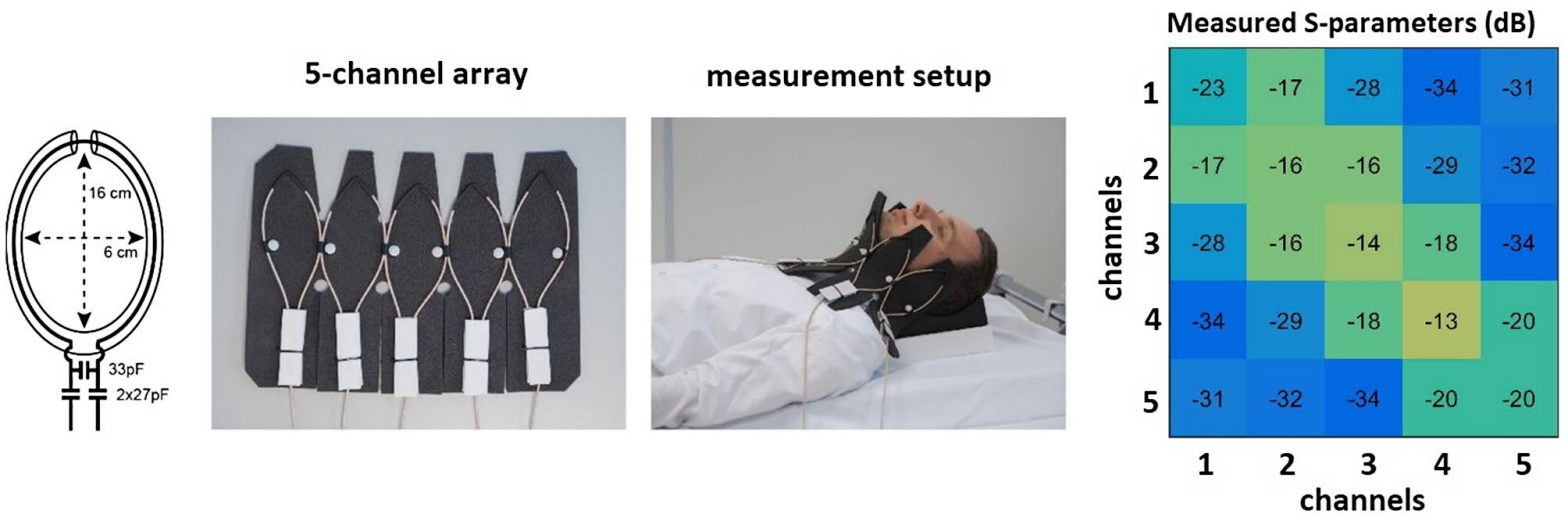
Coil schematics, fabricated five‐channel array, measurement setup on volunteer, and measured S‐parameters on a workbench

Each elongated transceive SCC loop coil (Figure 1A), with minor axis 60 mm and major axis 160 mm, was constructed from thin 50 Ohm coaxial cable (Huber+Suhner K 02252 D‐06, diameter 3.0 mm). Impedance matching used a balanced capacitive pi‐matching network: two 27‐pF high‐voltage‐rated (7200 V) capacitors (Dalicap, Dalian, China) were connected in series and one high‐voltage‐rated 33‐pF capacitor in parallel. The transceive array was constructed of five identical SCC loop coils spaced 5 mm apart (Figure 1). The elements were attached to a flexible foam former, with a thickness of 10 mm. The cable length connecting the coil and the interface was 1000 mm long. The array elements were tuned on a volunteer whose neck circumference was 370 mm because this was approximately the median dimension of the measured neck circumferences of five volunteers (two females and three males). Measured S‐parameters on a workbench are shown in Figure 1.

### 2.2 Electromagnetics simulations

Electromagnetic simulations of transmit efficiency and specific absorption rate (SAR) for the coil array at 298 MHz were performed in CST Microwave Studio 2019 (CST Studio Suite, Computer Simulation Technology, Darmstadt, Germany). To evaluate SAR_10g_ in vivo, simulations using the voxel model Gustav (with neck circumference ~375 mm and a voxel size of 2 × 2 × 2 mm) were used. Open boundary conditions were applied in all directions. The SCC coil is possible to simulate with frequency‐domain solver with tetrahedral meshing, as hexahedral meshing is not able to properly mesh the SCC coil.^8^ It is shown extensively in simulations and measurements^8^ that conventional and SCC coils exhibit the same properties regarding $B_{1}^{+}$ fields, SAR, and thermometry experiments. The voxel model is possible to simulate only with hexahedral meshing (time domain solver) and for that reason, SCC coils were simulated as conventional coils of the same geometry. As shown in,^8^ SCC elongated coil has the same characteristics in terms of $B_{1}^{+}$ and SAR as a conventional elongated coil with three distributed capacitors. In simulations, we tuned and matched every channel individually. The values of the matching capacitors were around 137 pF, tuning capacitors were around 23 pF, and three distributed capacitors on each coil had a value of 2.1 pF. Different excitation phases, corresponding to $B_{1}^{+}$ shimming, were investigated. The calculated SAR_10g_ was normalized to 1 Watt of accepted power. The minimum mesh size cell used was 1.2 mm, the number of mesh cells was around 11 million and simulations took approximately 60 minutes on a dual Intel Xeon x5660 CPU system and two NVIDIA K40C GPUs with 192 Gb DDR3 memory.

### 2.3 MRI measurements

All experiments were performed on a Philips Achieva whole‐body 7 Tesla scanner equipped with multiple transmit/receive channel capability, with fully independent magnitude and phase control on each channel. For in vivo experiments, informed consent was obtained from each of the healthy volunteers under a locally approved institutional review board protocol.

Transmit field ( $B_{1}^{+}$) maps were obtained using the dual refocusing echo acquisition mode (DREAM)^9^ sequence with the following parameters: field‐of‐view (FOV) = 400 × 320 × 25 mm^3^, voxel size = 5 × 5 × 5 mm^3^, flip angle = 10°, stimulated echo acquisition mode (STEAM) angle = 50°, TE/TR = 1.97/15 ms, 1 signal average.

Based on the in vivo $B_{1}^{+}$ maps, phase‐based RF shimming was performed using five individual transmit channels in order to optimize the transmit homogeneity within both carotids simultaneously. The vendor‐supplied optimization algorithm was used to optimize the transmit phases: equal magnitude excitation was used on all channels.

Anatomical localizer imaging of the carotids was performed with a gradient echo sequence using the following parameters: FOV = 170 × 170 mm^2^, voxel size = 0.6 × 0.6 × 4.0 mm^3^, flip angle = 25°, TR/TE = 300 /6.4 ms, number of averages = 4.

Black‐blood imaging at different spatial resolutions was performed with a multislice (MS) turbo spin echo (TSE) sequence (adapted from^5^) using the following common parameters––number of slices = 5, FOV = 140 × 140 mm^2^, echo train length = 12, number of averages = 1 and with pulse triggering:

- voxel size = 1 × 1 × 2.0 mm^3^ ‒ TE/TR = 7.9/4958 ms,
- voxel size = 0.4 × 0.4 × 2.0 mm^3^ ‒ TE/TR = 9.8/5240 ms, and
- voxel size = 0.25 × 0.25 × 2.0 mm^3^ ‒ TE/TR = 11/2622 ms.

## 3 RESULTS

Figure 1 shows the measurement setup and measured S parameters on a workbench for a neck circumference of 370 mm (for which the array was tuned). The measured s parameters values were −13 dB and better.

**Figure 2:**
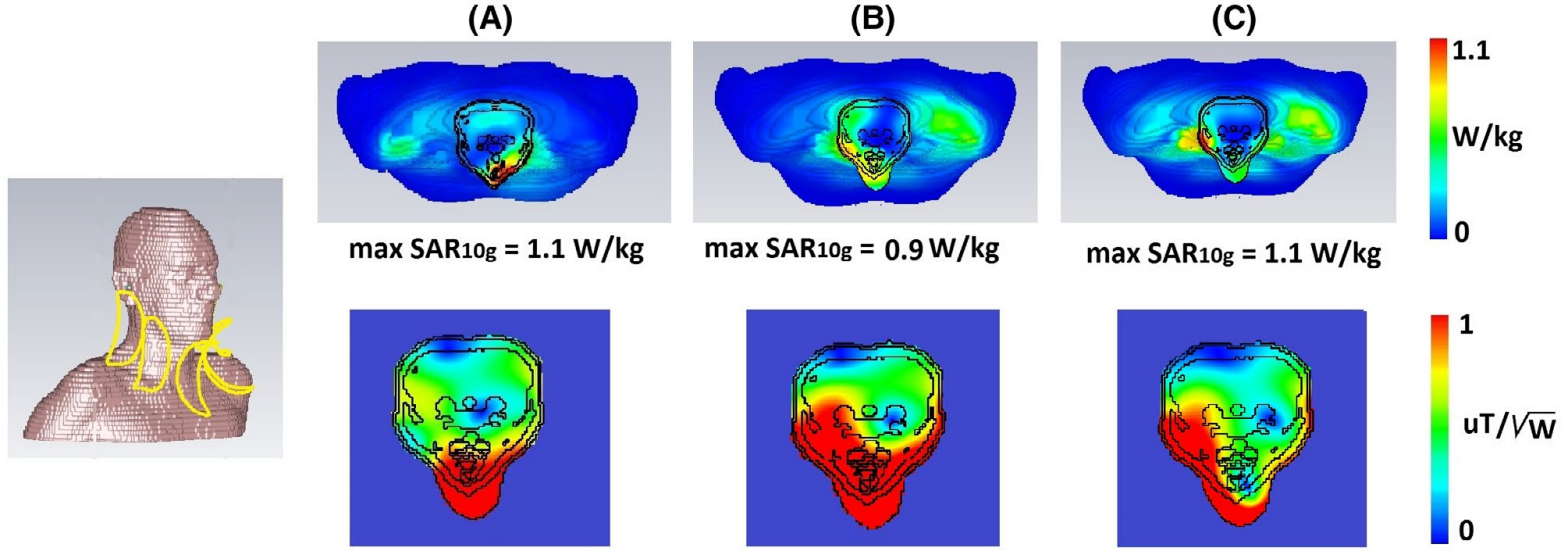
Simulated SAR_10g_ on a voxel model (upper row) and corresponding $B_{1}^{+}$ maps (lower row). (A) All phases were zero, (B) in vivo phase setting for both carotids visualization, and (C) in vivo phase setting for visualization of one carotid. Maximum simulated SAR_10g_ was 1.1 W/kg

In simulations, the coupling between the adjacent coils was −9.6 dB (except between the channels 3 and 4 where the coupling was −6.5 dB), and the reflection coefficients of all channels were at least −10 dB and resonance splitting has not been observed. Figure 2 shows the results from the SAR simulations and corresponding $B_{1}^{+}$ maps. As described in the methods, a number of different relative transmit phases were simulated and the results shown in Figure 2 correspond to the maximum peak SAR_10g_ value for three different situations––(1) all phases are 0, (2) phase setting is for visualization of both carotids (phases were 0°, 66°, 178°, −99° and −19°), and (3) phase setting is for the visualization of one carotid (phases were 0°, 110°, −38°, −148° and −42°). As reported in other work,^10^ the SAR is asymmetrically distributed within the neck region even for equal excitation phases, despite the symmetric distribution of transmit elements, due to the nonsymmetric nature of induced eddy currents within the body.^11^ The results show a maximum simulated SAR_10g_ of 1.1 W/kg on the voxel model (SAR_10g_ simulations have been normalized to 1 W of accepted power). Using a SAR limit of 10 W/kg,^12^ the maximum allowed input power is 9 W.

**Figure 3:**
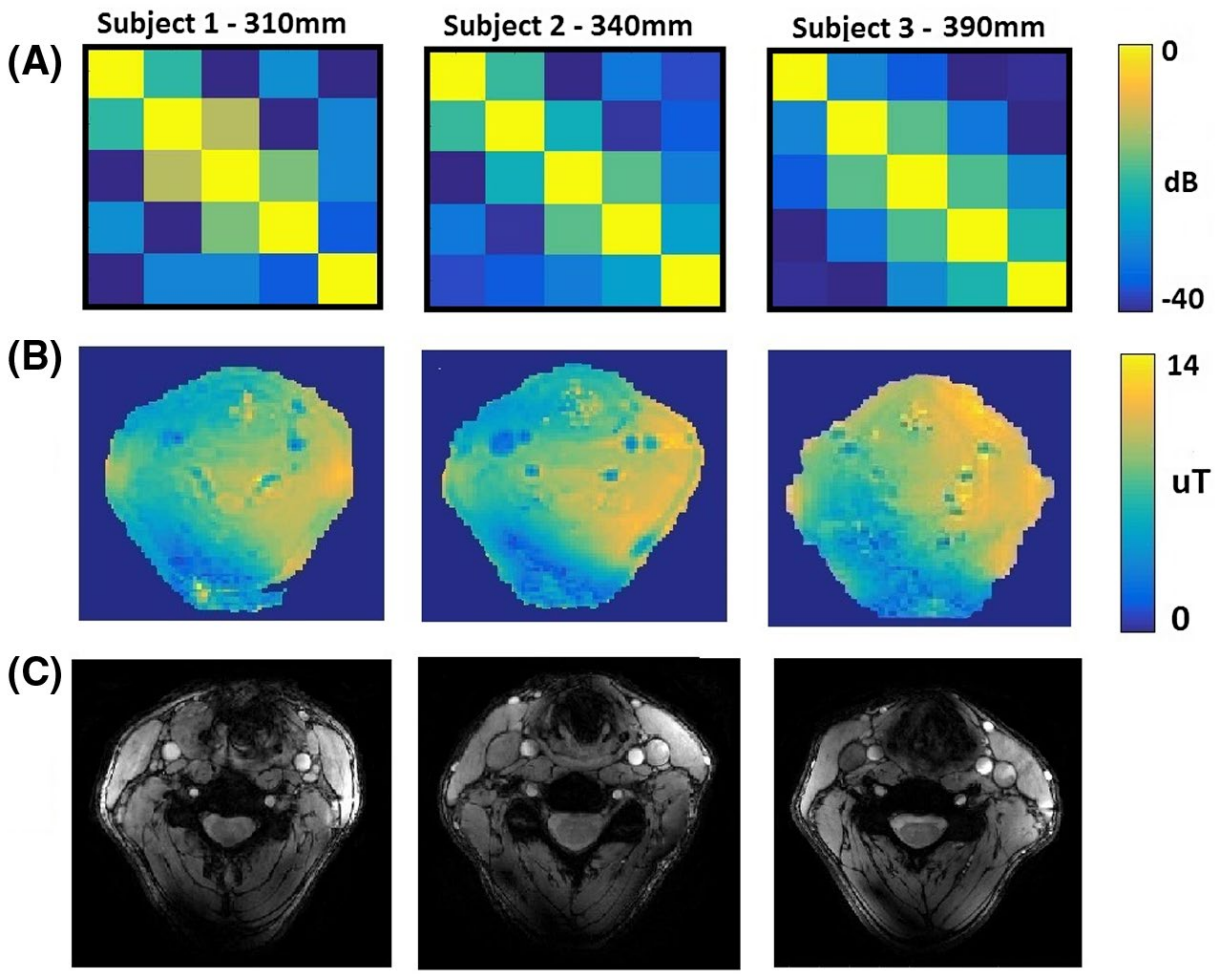
(A) Measured in vivo noise correlation matrices on subjects with different neck circumferences. (B) Measured $B_{1}^{+}$ maps on subjects with different neck circumferences. (C) gradient recalled echo (GRE) images to show the visualization of carotids

Figure 3A shows measured noise correlation matrices on three subjects (1 male, 2 females) with neck circumferences of 310 mm, 340 mm, and 390 mm. The measured coupling coefficients were below −12.5 dB for all subjects and all coils, with no retuning between measurements. Figure 3B shows the measured $B_{1}^{+}$ maps based on the DREAM measurements. The $B_{1}^{+}$ in a carotid regions were between 12.6 µT and 16.8 µT (between 90% and 120% of the target $B_{1}^{+}$ value, which was 14 µT, has been achieved) for each volunteer and for both carotids (note that these represent interpolated values since the $B_{1}^{+}$ in the carotid arteries themselves cannot be measured due to flow). Figure 3C shows anatomical localizer gradient echo images of the carotids, with the carotids appearing bright due to in‐flow effects.

**Figure 4:**
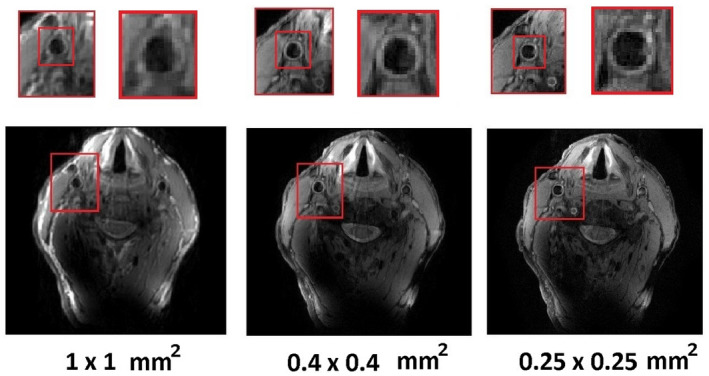
Measured turbo spin echo (TSE) carotid images in three different resolutions. The highest resolution achieved was 0.25 × 0.25 mm^2^ (the slice thickness was 2 mm in all cases)

Figure 4 shows ‘’black‐blood’’ TSE images of the carotid artery for one of the three volunteers at three different in‐plane spatial resolutions (1 × 1 mm^2^, 0.4 × 0.4 mm^2^, and 0.25 × 0.25 mm^2^), with zoomed‐in magnifications to show the excellent depiction of the vessel wall.

## 4 DISCUSSION

Previous work has shown that an SCC coil has high intrinsic decoupling from surrounding elements and that the bending and elongation of the coil does not influence its performance as much as the conventional loop coil^8^ making it very suitable for a flexible array. Bending and elongation of the SCC coil also does not affect maximum SAR_10g_ values. The asymmetry of $B_{1}^{+}$ field is present when RF shimming has been performed for the visualization of both carotids. That can be attributed to the induced conductive currents that affect the phase of the $B_{1}^{+}$ field.^11^ In some subjects, the mentioned asymmetry prevents the visualization of both carotids at the same time. Visualization of both carotids at the same time would have high diagnostic relevance because it would allow side‐by‐side comparison of the arteries. The presence of asymmetry can be resolved by adding three more SCC elements on the back side of the neck. The new eight‐element array completely surrounds neck and initial phantom and in vivo experiments (not shown here) did not have asymmetry present. The future work will further explore performance of the 8‐element array. The coil flexibility was demonstrated by imaging of three subjects with very different neck circumferences, with the measured noise correlation coefficients being stable with respect to the neck dimensions.

## 5 CONCLUSIONS

In this paper, we designed a highly flexible 5‐element array for imaging of the carotid arteries at 7 T. The fabricated array can be positioned tightly onto the anterior neck region and can be bent to follow the anatomy of the region. Using this array we have achieved very high resolution, 0.25 × 0.25 mm^2^ in‐plane black‐blood carotid imaging in a single acquisition.
